# Biofortified tomatoes provide a new route to vitamin D sufficiency

**DOI:** 10.1038/s41477-022-01154-6

**Published:** 2022-05-23

**Authors:** Jie Li, Aurelia Scarano, Nestor Mora Gonzalez, Fabio D’Orso, Yajuan Yue, Krisztian Nemeth, Gerhard Saalbach, Lionel Hill, Carlo de Oliveira Martins, Rolando Moran, Angelo Santino, Cathie Martin

**Affiliations:** 1 John Innes Centre, Norwich Research Park, Norwich, UK; 2 Institute of Sciences of Food Production, C.N.R., Unit of Lecce, Lecce, Italy; 3 Recombinant Biopharmaceutical Laboratory, Department of Pharmacology, Biological Sciences Faculty, University of Concepción, Concepción, Chile; 4 CREA—Research Centre for Genomics and Bioinformatics, Rome, Italy; 5 University of Glasgow, Glasgow, UK; 6 University of Camagüey, Camagüey, Cuba

**Keywords:** Molecular engineering in plants, Secondary metabolism

## Abstract

Poor vitamin D status is a global health problem; insufficiency underpins higher risk of cancer, neurocognitive decline and all-cause mortality. Most foods contain little vitamin D and plants are very poor sources. We have engineered the accumulation of provitamin D_3_ in tomato by genome editing, modifying a duplicated section of phytosterol biosynthesis in Solanaceous plants, to provide a biofortified food with the added possibility of supplement production from waste material.

## Abstract (web summary)

Vitamin D insufficiency is a major public health problem requiring dietary fortification and supplement solutions. This study produced gene-edited tomato lines that accumulate provitamin D_3_ in fruits, offering a new dietary source of vitamin D_3_.

## Main

Vitamin D prevents deficiency diseases affecting skeletal development^1^ and is converted to products with steroid hormone bioactivities, which function in signalling in many organs including the brain (Supplementary Fig. 1)^2^. Consequently, deficiencies in vitamin D impact immune function and inflammation and are associated with increased risk of micronutrient deficiencies^1^, cancer^3^, Parkinson’s disease^4^, depression^5^, neurocognitive decline^6^, dementia^7^ and the severity of coronavirus disease 2019 (COVID-19) infection^8^. Vitamin D can be synthesized by humans from 7-dehydrocholesterol (7-DHC), also known as provitamin D_3_, following exposure of skin to ultraviolet B (UVB) light^9^, but the major source is dietary^10^. Approximately one billion people worldwide suffer from vitamin D insufficiency^11^, and numbers are increasing largely because of inadequate dietary availability (Supplementary Fig. 1a). We have developed a new dietary source of vitamin D in plants to meet the increasing demand for ways to address vitamin D insufficiency, which is of particular relevance to those adopting plant-rich, vegetarian or vegan diets^10^.

The original identification of vitamin D_2_ in plants was eventually shown to be due to fungal infection^12^. 7-DHC is synthesized by some plants such as tomato, on route to cholesterol and steroidal glycoalkaloid (SGA) synthesis, predominantly in leaves. UVB exposure of leaves of tomato produces vitamin D_3_ (ref. ^13^). However, plants are generally poor dietary sources of vitamin D_3_, and the best are fish and dairy products. Mushrooms and yeast can be used as sources of vitamin D_2_, following exposure to UVB light, but vitamin D_2_ has been reported to be substantially less bioeffective than vitamin D_3_ in several epidemiological studies^14^.

**Fig. 1:**
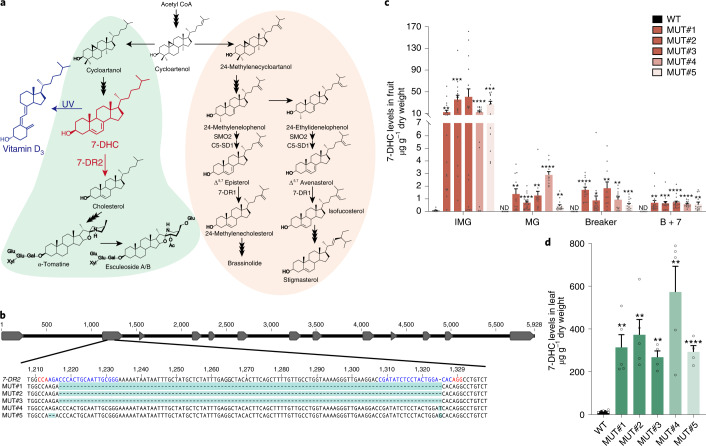
7-DHC accumulated in *Sl7-DR2*-homozygous-knockout lines. **a**, The cholesterogenesis pathway (depicted in light green) and phytosterol biosynthesis pathway (depicted in light orange) in tomato, redrawn from Sonawane et al.^16^. 7-DHC is converted by 7-DR2 to cholesterol, which can be converted to vitamin D_3_ by exposure to UVB light. SMO, C-4 sterol methyl oxidase; C5-SD1, sterol C-5(6) desaturase 1. **b**, Five independent *Sl7-DR2*-knockout lines were generated by genome editing. Top: schematic structure of *Sl7-DR2* gene, with exons indicated as grey arrows. Bottom: recovered mutations in each line are highlighted in light blue. The CRISPR–Cas9-targeted sequences and the protospacer-adjacent motif sequences are shown in blue and red, respectively. **c**, 7-DHC contents in wild-type (WT) and *Sl7-DR2*-knockout tomato fruit at different stages of ripening (IMG, immature green; MG, mature green; Breaker, fruit turning ripe; B + 7, 7 days after breaker-ripe fruit). Data are presented as the mean ± s.e.m. From left to right: *n* = 14, 19, 16, 15, 16, 14, 13, 11, 18, 13, 10, 15, 15, 14, 17, 11, 11, 15, 9, 17, 14, 17, 15 and 15 biologically independent fruit samples. ND, not detected. **d**, 7-DHC content of leaves of wild-type and *Sl7-DR2*-knockout lines. Data are presented as mean ± s.e.m. From left to right: *n* = 4, 5, 5, 4, 5 and 4 biologically independent leaf samples. Statistical significance between WT and mutants at each fruit ripening stage (**c**) or in leaves (**d**) was assessed using two-tailed *t*-tests (*P ≤ 0.05, **P ≤ 0.01, ***P ≤ 0.001, ****P ≤ 0.0001). Source data

Although 7-DHC has been identified in tomato leaves, it does not normally accumulate in fruit, where it serves as an intermediate in the formation of SGAs: tomatines in green fruit and esculeosides in ripe fruit^13,15^. Recently it has been shown that a duplicate pathway operates in Solanaceous species, including tomato, where specific isoforms of some enzymes, that are generally responsible for phytosterol and brassinosteroid biosynthesis, produce cholesterol for the formation of SGAs^16^ (Fig. 1a). This partial separation of phytosterol and cholesterol biosynthesis allows metabolic flexibility for the synthesis of important hormones (brassinosteroids) and more specialized stress chemicals, such as SGAs, with fungicidal, antimicrobial and insecticidal properties^16^. The existence of a ‘duplicate’ pathway for SGA biosynthesis in tomato makes the engineering of 7-DHC accumulation relatively straightforward. A specific isoform of 7-dehydrocholesterol reductase (Sl7-DR2) converts 7-DHC to cholesterol for the synthesis of α-tomatine in leaves and fruit^16^ (Fig. 1a). Consequently, knocking out the activity of Sl7-DR2 should result in the accumulation of 7-DHC with minimal impact on phytosterol and brassinosteroid biosynthesis. With the aim of biofortifying tomato in provitamin D_3_, we tested the efficacy of blocking Sl7-DR2 activity using clustered regularly interspaced short palindromic repeats–CRISPR-associated protein 9 (CRISPR–Cas9) genome editing to increase 7-DHC levels. Two single-guide RNAs (sgRNAs) were designed to sequences within the second exon of the *Sl7-DR2* gene (Fig. 1b), with minimal homology between the sgRNAs and the only other sterol Δ^7^ reductase gene in tomato (*Sl7-DR1*). We recovered five independent knockout alleles of the *Sl7-DR2* gene within the T1 generation, three of which carried identical deletions of 108 bp of the exon 2 sequence between the two sgRNAs (MUT#1, MUT#2 and MUT#3). Two other knockout alleles were created by deletion of 2 bp with insertion of 1 bp (MUT#5) or by insertion of 1 bp only in the second exon (MUT#4), both of which caused frame shifts and predicted premature termination of the Sl7-DR2 protein (Fig. 1b). Homozygous-knockout alleles were recovered in the T1 generation, and homozygous-knockout lines lacking the transfer DNA (T-DNA) carrying the *Cas9* gene and the sgRNA sequences were recovered for four of the five lines within the T2 generation.

Five homozygous-knockout alleles of *Sl7-DR2* were selected, following segregation, in the T2 generation. No off-target edits of the *Sl7-DR1* gene were detected in these mutant lines (Supplementary Fig. 3). Fruit and leaves were analysed for 7-DHC content as well as levels of other phytosterols, cholesterol and SGAs using liquid chromatography–mass spectrometry (LC–MS)^13,17^.

**Extended Data Fig. 1:**
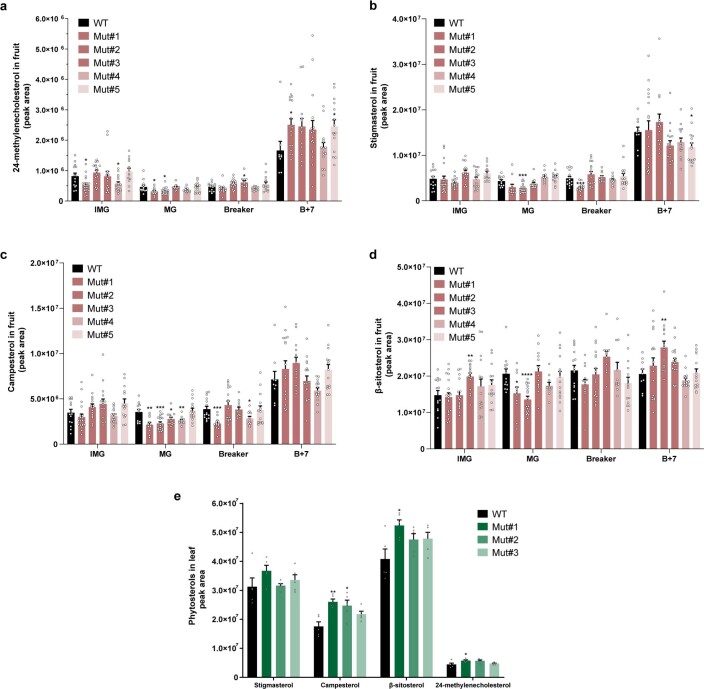
Relative contents of four representative phytosterols (stigmasterol, campesterol, β-sitosterol and 24-methylenecholesterol) of wild-type (WT) and *Sl7-DR2* knock-out fruit during ripening (a-d) and leaves (e). (mean ± s.e.m). **a-d**, from left to right, n = 14, 19, 16, 15, 16, 14, 13, 11, 18, 13, 10, 15, 15, 14, 17, 11, 11, 15, 9, 17, 14, 17, 15, and 15 biologically independent fruit samples; **e**, n = 5 biologically independent leaf samples for each line. Statistical significance between WT and mutants at each fruit ripening stage (**a-d**) in leaves (**e**) was assessed using two-tailed *t*-tests (*P ≤ 0.05; **P ≤ 0.01; ***P ≤ 0.001; ****P ≤ 0.0001). See source data for *P* values where relevant. Source data

Loss of Sl7-DR2 activity had no effect on the growth, development or yield of the tomato lines (Supplementary Fig. 1c,d). This contrasts with the phenotype of the loss-of-function mutation of the gene encoding sterol Δ^7^-reductase involved in phytosterol biosynthesis in *Arabidopsis* (*DWARF5*), which is dwarfed because of an inhibition of brassinosteroid biosynthesis^18^. The lack of effect of mutations in *Sl7-DR2* on phytosterol metabolism was confirmed by comparing the levels of stigmasterol, an end product of the phytosterol pathway in tomato, in leaves of wild-type and edited lines (Supplementary Fig. 1e) and other phytosterols in fruit and leaves (Extended Data Fig. 1). In wild-type plants, 7-DHC was detected only in immature green fruit and was undetectable in ripening and ripe fruit. In contrast, loss of Sl7-DR2 activity resulted in substantial increases in 7-DHC levels in leaves and green fruit (Fig. 1c,d). Levels of 7-DHC were lower in ripe fruit of the *Sl7-DR2* mutants (Fig. 1c) but remained high enough that, if converted to vitamin D_3_ by treatment with UVB, the amount in one tomato would be equivalent to that in two medium-sized eggs or 28 g of tuna, which are both recommended dietary sources of vitamin D (FoodData Central USDA, https://fdc.nal.usda.gov/). For the elderly with declining levels of 7-DHC, consuming fruit biofortified with 7-DHC might address their deficiencies directly^19^.

**Fig. 2:**
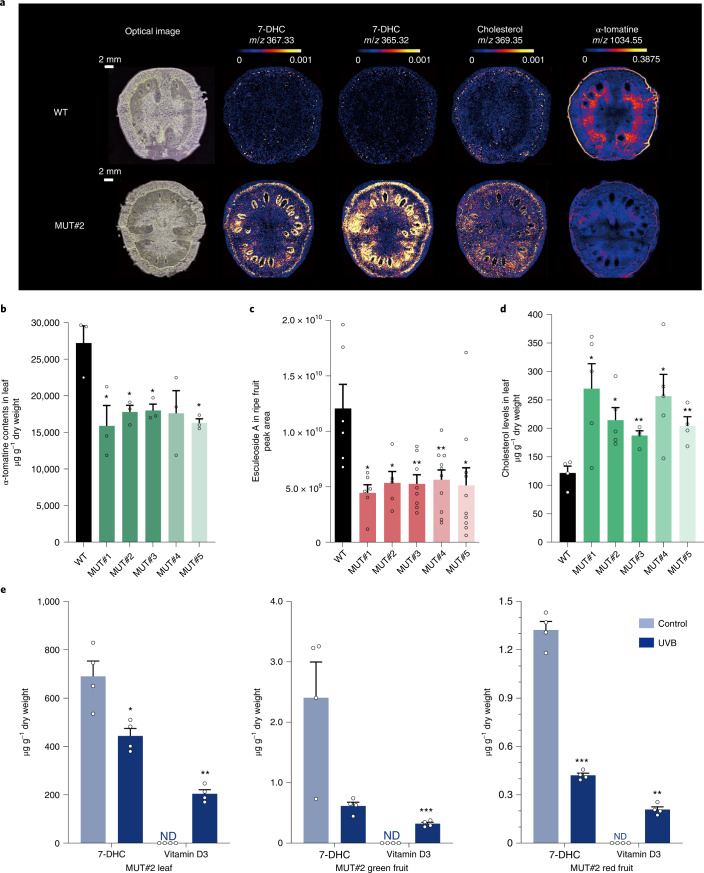
Localization and quantitative comparison of 7-DHC, SGAs and cholesterol in wild-type and *7-DR2*-knockout lines and conversion of 7-DHC in *Sl7-DR2* knockouts to vitamin D_3_ by UVB irradiation. **a**, MALDI images of 7-DHC (*m*/*z* 367.33) and its laser-induced derivative ion (*m*/*z* 365.32), cholesterol (*m*/*z* 369.35) and α-tomatine (*m*/*z* 1,034.55). Scale bar, 2 mm. The HotMetal2 colour scale indicates the range of total ion current-normalized intensity. The same metabolite is shown with identical scale intensity for wild-type and mutant samples. It is not straightforward to compare the relative abundance of different metabolites using MALDI images due to potentially different ionization efficiencies. **b**, α-Tomatine contents of leaves of wild-type and *Sl7-DR2*-knockout lines (mean ± s.e.m, *n* = 3 biologically independent leaf samples for each line). **c**, Relative esculeoside A content of red-ripe (seven days after breaker) fruit of wild-type and *Sl7-DR2*-knockout lines (mean ± s.e.m). From left to right: *n* = 6, 6, 5, 8, 10 and 10 biologically independent fruit samples. **d**, Cholesterol content of leaves of wild-type and *Sl7-DR2*-knockout lines (mean ± s.e.m). From left to right, *n* = 4, 5, 5, 4, 5 and 4 biologically independent leaf samples. **e**, Contents of 7-DHC and vitamin D_3_ in control and UVB-treated leaves or fruit (mean ± s.e.m, *n* = 4 biologically independent leaf or fruit samples at each stage for control and MUT#2). Tissues of Mut#2 were irradiated by UVB light for 1 h. The experiment was repeated three times. ND, not detected. Statistical significance between WT and mutant values (**b**–**d**) and between control and UVB-treated tissue (**e**) was assessed using two-tailed *t*-tests (*P ≤ 0.05, **P ≤ 0.01, ***P ≤ 0.001, ****P ≤ 0.0001). Source data

Matrix-assisted laser desorption/ionization (MALDI) imaging showed that the increases in 7-DHC were distributed in both the flesh and peel of tomatoes (Fig. 2a and Supplementary Fig. 1f). α-Tomatine and dehydrotomatine are converted to esculeoside A and dehydroesculeoside A, respectively, during fruit ripening, meaning that tomatines are reduced to low levels in ripe fruit^20^. MALDI imaging of mutant and wild-type green fruit showed that α-tomatine was lower in the *Sl7-DR2* mutants than in controls (Fig. 2a and Supplementary Fig. 1f,g), and leaves showed substantially lower levels in the mutants, although α-tomatine was not eliminated (Fig. 2b). A strong reduction in the levels of the SGA, esculeoside A, was also observed in ripe fruit of the mutants compared with the control (Fig. 2c). The reduction in α-tomatine might be considered beneficial because of its reported toxicant/antinutritional activity. Interestingly, cholesterol levels were generally higher than in wild-type controls in both fruit (Fig. 2a and Supplementary Fig. 1h) and leaves (Fig. 2d). This suggested that the block in flux along the SGA biosynthetic pathway may be compensated by increased flux of intermediates, perhaps catalysed by the enzymes of the phytosterol pathway (or at least Sl7-DR1), which supplement cholesterol production and limit reductions in SGA accumulation. However, this does not involve compensatory changes in expression of the genes encoding enzymes in either pathway as shown by reverse transcription quantitative PCR (RT–qPCR) analysis of these genes in leaves of wild-type and mutant lines (Supplementary Fig. 2a). Only *SlC5-SD1* in the phytosterol pathway showed consistently lower (~30%) transcript levels than controls (Fig. 1a and Supplementary Fig. 2b).

As an important confirmation of our biofortification strategy, we tested whether the elevated levels of 7-DHC in *Sl7-DR2*-mutant plants could be converted to vitamin D_3_ by irradiating leaves and sliced fruit with UVB light for 1 h as described by Jäpelt et al.^17^. Treatment of leaves was very effective, resulting in yields of vitamin D_3_ of nearly 200 μg g^−1^ dry weight (Fig. 2e). Yields from fruit were lower, reaching 0.3 μg g^−1^ dry weight in green fruit and 0.2 μg g^−1^ dry weight in red fruit, reflecting the declining content of 7-DHC in green fruit and red-ripe fruit compared with leaves (Fig. 2e). A medium-size tomato has a dry weight of about 8–10 g, and the levels of vitamin D_3_ that could be achieved in one *Sl7-DR2*-mutant tomato approach 30% in green fruit and 20% in red fruit of the recommended daily allowance (RDA) calculated on baseline dietary intakes of 2.8 μg per day (United Kingdom) and 4.3 μg per day (United States)^21,22^. Vitamin D_3_ in ripe fruit might be increased further by extended exposure to UVB, for example, during sun-drying.

The duplicate pathway for cholesterol/SGA biosynthesis exists in other food crops of the Solanaceae family, including eggplant (*Solanum melongena*), potato (*Solanum tuberosum*) and pepper (*Capsicum annuum*)^16^. The close association between cholesterol/SGA biosynthesis, 7-DHC accumulation and photosynthesis in leaves and green fruit of tomato (Figs. 1c,d and 2b–d and Supplementary Fig. 1g,h) suggests that knockouts of 7-DR2 activity in pepper, where fruit may be green when eaten, might also provide a vitamin D_3_-biofortified, plant-based food. Mutations that increase UVB penetration into fresh fruit, such as *y* in tomato that causes the loss of UV-protecting chalcones from the skin of ‘pink tomatoes’, might offer increased UVB conversion of provitamin D_3_ to vitamin D_3_. Such stacking could be achieved by further gene editing or by introgression^23^. The leaves of the *Sl7-DR2* mutants are rich sources of provitamin D_3_ and consequently could provide an important new feed stock using the waste vegetative material from tomato cultivation for the manufacture of vitamin D_3_ supplements from plants that would be suitable for vegans. Editing of *Sl7-DR2* could generate similar alterations in any elite tomato variety, meaning that tomato could be developed as a plant-based, sustainable source of vitamin D_3_.

## Methods

### Plant materials

Tomato (*Solanum lycopersicum*) cv. Money Maker and knockout mutants of *Sl7-DR2* were grown in the greenhouse at an average ambient temperature of 20–22 °C. Supplemental lighting was available to maintain 16 h of light per day when necessary.

### Plasmid construction

Two specific target sequences (Fig. 1b) in exon 2 of the *Sl7-DR2* (*Solyc06g074090*) gene were selected to generate *Sl7-DR2*-knockout mutants. These were introduced into the sgRNA scaffold by PCR. To make the sgRNA-expression cassette, each sgRNA amplicon and a synthesized U6-III promoter (pICSL90001) were cloned into a GoldenGate Level 1 acceptor (pICH47732 and pICH47742). A Level 2 binary vector, pICSL002203, containing the Cas9-expression cassette and kanamycin-resistance-expression (nptII) cassette, was used as the destination vector to generate the *Sl7-DR2*–CRISPR–Cas9 construct. sgRNA efficiency was tested by co-transformation of tomato using *Agrobacterium rhizogenes* (strain ArATCC15834)^24^. The sequences of exon 2 of *Sl7-DR2* were amplified by PCR directly from hairy roots with the Phire Plant Direct PCR Master Mix following the manufacturer’s instructions (Thermo Scientific) using primers flanking the sgRNA target sequences (forward: TGTTTCACTGGGCTGGTTTAGC and reverse: GAGAAGTCTTTCACCATGTCACGA). Stable transformations were conducted after the sgRNA efficiency check^25^.

### Tomato stable transformation

The *Sl7-DR2*–CRISPR–Cas9 construct was transformed into *Agrobacterium tumefaciens* (strain AGL1) for stable transformation, which was undertaken using cotyledons as initial explants.

### Screening of *Sl7-DR2*-knockout lines

DNA was isolated from the finely ground powder of leaf tissues using DNeasy Plant Mini Kits (Qiagen) following the manufacturer’s instructions. Five independent *Sl7-DR2*-knockout lines were obtained by genotyping with primers flanking the sgRNA target sequences (forward: TGTTTCACTGGGCTGGTTTAGC and reverse: GAGAAGTCTTTCACCATGTCACGA) and confirmed by sequencing.

### RT–qPCR analysis

Total RNA was extracted from tomato leaf tissues using the Trizol method (Sigma-Aldrich). DNase I (Roche)-treated RNA was reverse transcribed using SuperScript™ III (Invitrogen). SYBR Green JumpStart Taq ReadyMix (Sigma-Aldrich) was used to perform all the RT–qPCR reactions using the X96 Touch Real-Time PCR Detection System (Biorad). Data were analysed using CFX Maestro Software. *SlActin* (*Solyc03g078400*) was selected as the house-keeping reference gene. The relative expression of genes was calculated by the ΔCt method. Gene-specific primers were designed using National Center for Biotechnology Information (NCBI) primer Basic Local Alignment Search Tool (BLAST) (https://www.ncbi.nlm.nih.gov/tools/primer-blast/), listed in Supplementary Table 1. All primers used in this study were synthesized by Sigma-Aldrich.

### MALDI-imaging analysis

Cryosectioning of fruit was undertaken as described by Dong et al.^26^. Fresh immature green fruit (about 16 days after anthesis) was flash frozen in liquid nitrogen and then embedded with M1 embedding matrix (Thermo Scientific) on a flat metal holder on dry ice. The embedded tissues were transferred to a CryoStar NX70 Microtome (Thermo Scientific) and thermally equilibrated at −18 °C for at least 3 h. The tissues were cut into 35-µm-thick sections and thaw-mounted on Superfrost Plus slides (Thermo Scientific), followed by vacuum drying in a desiccator.

Optical images were taken using a Canon 5D Mark IV camera with a Canon MP-E 65 mm f/2.8 1–5x Macro Photo lens (Canon) at 1:1 ratio. Raw image files were processed with Capture One photo editing software (Capture One).

Sections were covered with 2,5-dihydroxybenzoic acid matrix (DHB) using a SunCollect MALDI Sprayer (SunChrome) with a DHB solution of 10 mg ml^−1^ in 80% methanol/0.05% trifluoroacetic acid (TFA) to a density of approximately 3 µg mm^−2^.

MALDI imaging was performed with a Synapt G2-Si mass spectrometer with a MALDI source (Waters) equipped with a 2.5 kHz Nd:YAG (neodymium-doped yttrium aluminum garnet) laser operated at 355 nm. The slides were fixed in the instrument metal holder and were scanned with a flat-bed scanner (Canon). The images were used to generate pattern files and acquisition methods in the HDImaging software version 1.4 (Waters) with the following parameters: area of a complete section approximately 400 mm^2^, laser beam diameter at low setting (60 µm) with 105 µm step size, resulting in approximately 36,000 pixels per section, MALDI–MS-positive sensitivity mode, *m*/*z* 50–1,200, scan time 0.5 s, laser repetition rate 1 kHz, laser energy 200. For ion-mobility measurements, the same parameters were used in MALDI–high-definition mass spectrometry (HDMS) mode with the following additional tune page settings: trap d.c. bias, 45.0; transfer wave velocity (m s^−1^), 315; IMS (ion mobility spectrometry) wave height (V), 40.0; variable wave velocity enabled with linear ramp, wave velocity start (m s^−1^): 1,500.0; wave velocity end (m s^−1^), 200.0. Red phosphorous clusters were used for instrument calibration and lock mass correction. Total scan time for a complete section was 10–12 h, and the lock mass was acquired every 10 min for 2 s.

The MS raw files were processed in HDI1.4 with the following parameters: detection of the 2,000 most-abundant peaks, *m*/*z* window 0.05, MS resolution 10,000, lock mass 526.554 (red phosphorous cluster). The processed data were loaded into HDI1.4 and normalized by total ion content. Images were generated using the HotMetal2 colour scale and exported as png image files. Compounds of interest, 7-DHC, cholesterol and α-tomatine were identified by comparison with the drift time and mass of authentic standards analysed on the same instrument. The masses detected for 7-DHC, vitamin D_3_ and cholesterol during MALDI are listed in Supplementary Table 2. It has been reported that cholesterol is susceptible to laser-induced oxidation during MALDI time of flight MS^27^, and 7-DHC has an even higher tendency for non-enzymatic autoxidation^28,29^. Among the peaks of standards generated during MALDI, taking into account their specificity and relative abundance, 367.33, 365.32 and 363.31 were selected as representative masses for 7-DHC and 369.35 and 1034.55 were selected as representative masses for cholesterol and α-tomatine, respectively (Supplementary Table 2).

### Sterol analysis

The methods for sterol extraction and analysis were modified from Jäpelt et al.^14^. For consistency of extraction efficiency and comparability among samples, all extracts were prepared with the same mass of tissue per volume of extraction solvents. Freeze-dried material (about 20 mg) was weighed into a 2 ml Eppendorf tube and mixed with 100 µl 60% potassium hydroxide (Sigma-Aldrich), 500 µl 96% ethanol (Sigma-Aldrich) and 300 µl 15% ascorbic acid (Sigma-Aldrich). To avoid thermal isomerization of vitamin D_3_ to previtamin D_3_, we applied overnight cold saponification rather than hot saponification. The sample preparation process was carefully maintained at or below room temperature. The tubes were shaken for approximately 18 h at 22 °C in a thermoshaker (Eppendorf). Twenty percent ethyl acetate in pentane (v/v) (750 µl) was added and shaken for 30 min on a flat shaker, followed by centrifugation at 2000 × *g* for 5 min at room temperature. The organic layer was transferred into a new 2 ml Eppendorf tube. The extraction steps were repeated twice. Total extracts were washed with 500 µl of 0.1 mol l^−1^ hydrochloric acid by inverting tubes 30 times to completely remove the alkali. The upper layer was transferred into a 2 ml Eppendorf tube following centrifugation at 1000 × *g* for 2 min. Total extracts were evaporated to dryness using a Genevac EZ-2 Elite Evaporator with the programme of ‘Very Low BP Mix’. The residue was finally redissolved in 200 µl methanol and filtered through 0.22 μm nylon Corning® Costar® Spin-X® tube filter (Sigma-Aldrich). The samples were stored at −80 °C until analysis.

Sterol compounds were identified by comparison with the retention time and mass spectrometry spectra of authentic standards (Sigma-Aldrich) analysed on the same instrument, and were quantified based on calibration curves generated with authentic standards running along with the samples using Xcalibur Quan Brower software (Version 4.3). LC analysis was undertaken on a Dionex UltimMate (Thermo Scientific) equipped with a thermostated column compartment. The chromatographic separation was done on a 50 × 2.1 mm 2.6 μm Kinetex F5 column (Phenomenex) at a flow rate of 0.6 ml min^−1^. Solvents were 0.2% formic acid and 25% acetonitrile in Milli-Q water (v/v) (A) versus 100% methanol (B). The gradient programme was as follows: 60% B for 0.5 min, a linear gradient to 85% B for 7 min, a linear gradient to 100% B for 0.5 min, isocratic elution for 1 min and 0.5 min linear gradient back to 60% B and re-equilibration for 3.5 min, giving a total run time of 13 min. The column was maintained at 40 °C. Five microlitre samples were injected. MS was performed using a Q Exactive Orbitrap Mass Spectrometer (Thermo Scientific) with an atmospheric pressure chemical ionization source. The MS was set up to collect full scans at 70,000 resolution from *m*/*z* 180–2,000 and data-dependent MS2 of the top 4 ions, at an isolation width of *m*/*z* 4.0, 30% normalized collision energy. These ions were then ignored for 5 s in favour of the next-most-abundant ion; isotope peaks were also ignored. Data-dependent MS2 analysis was at 17,500 resolution, maximum ion time of 50 ms, automatic gain control target of 1 × 105 ions. MS scans had a maximum ion time of 50 ms and automatic gain control target of 3 × 106 ions. Spray chamber conditions were 231 °C capillary temperature, 21.25 units sheath gas, 5 units aux gas, no spare gas, 4 μA current, 363 °C probe heater temperature and 50V S-lens RF. Samples were run in randomized order to avoid systematic bias. Xcalibur software (version 4.3, Thermo Scientific) was used for instrument control and data acquisition.

### SGA analysis

SGA extraction was performed as described by Itkin et al.^30^. Briefly, 20 mg of freeze-dried samples (leaf or fruit) were sonicated in 1 ml 100% methanol, incubated on ice for 1 h and centrifuged at 1,700 × *g* for 10 min. The supernatants were collected, centrifuged at 1,700 × *g* for 3 min and filtered with a 0.22 μm filter. The sample extracts were stored at −80 °C until the analysis. α-tomatine was identified and quantified by comparing the retention time and MS spectrum to the authentic standard (Sigma-Aldrich). Esculeoside A was identified on the basis of its MS spectrum compared with previously published spectra and relative retention time.

Chromatographic separation was performed on a 50 × 2.1 mm 2.6 μm Kinetex EVO C18 column (Phenomenex) at a flow rate of 0.6 ml min^−1^. Solvents were 0.1% formic acid in Milli-Q water (v/v) (A) versus 100% acetonitrile (B). The gradient programme was as follows: a linear gradient from 2% B to 40% B for 4 min, a linear gradient to 95% B for 2 min, isocratic elution for 1 min and a 0.1 min linear gradient back to 2% B and re-equilibration for 2.1 min, giving a total run time of 9.2 min. MS was performed using a Q Exactive Orbitrap Mass Spectrometer (Thermo Scientific) with an electrospray ionization (ESI) source. All other settings were the same as those described above for sterol analysis.

### UV treatment

Fruits were cut into 1 mm slices before UV exposure. Leaf or fruit tissues were exposed to UVB light (3.2 mW cm^−2^) for 1 h at 20 cm below the inverted ultraviolet transilluminator (302 nm; Analytik-Jena). Samples were immediately frozen in liquid nitrogen after treatment for subsequent analysis.

### Statistical analysis

All the experiments in this paper were repeated at least three times independently, and results from representative datasets are presented. All numerical values are presented as means ± s.e.m. GraphPad Prism (version 9.2.0) was used for the statistical analysis. Statistical differences between wild-type and mutants were calculated using two-tailed *t*-tests with or without Welch’s correction, for comparing groups with or without unequal variance, respectively. Related information is listed in the Source Data.

### Reporting Summary

Further information on research design is available in the Nature Research Reporting Summary linked to this article.

## Supplementary information

Supplementary Information Supplementary Figs 1–3 and Tables 1 and 2.

Reporting Summary

Supplementary Data 1 Statistical source data for supplementary figures.

Supplementary Data 2 ChemDraw data.

## Data Availability

Materials generated and analysed in this study are available from the corresponding author upon request. A reporting summary for this paper is available as a Supplementary information file. Source data are provided with this paper.

## Source data

Source Data Fig. 1 Statistical source data.
Source Data Fig. 2 Statistical source data.
Source Data Extended Data Fig. 1 Statistical source data.
