# Supplementary material for: Biofortified tomatoes provide a new route to vitamin D sufficiency
Source: Nat Plants. 2022 May 23;8(6):611–6. doi: 10.1038/s41477-022-01154-6 (PMC9213236; doi:10.1038/s41477-022-01154-6)
Supplement: Supplementary file 1 — Supplementary Figs 1–3 and Tables 1 and 2. [file 41477_2022_1154_MOESM1_ESM.pdf]

nature portfolio

https://doi.org/10.1038/s41477-022-01154-6

Supplementary information

Biofortified tomatoes provide a new route to vitamin D sufficiency

In the format provided by the authors and unedited

a

Geographical area	Prevalence (%)	
	Cutoff of <25nmol/L or <30 nmol/L	Cutoff of <50nmol/L
Global[31]	6.7%	37.3%
US[32]	5.9%	24.0%
Canada[33]	7.4%	36.8%
Europe[34]	13.0%	40.4%
Afghanistan[35]	64.7%* (<20nmol/L)	95.5%*
China (Mainland)[36]	20.7%	63.2%
Japan[37]	NA	53.6%
India[38,39]	19.4-87%	67.0%
Iran and Jordan[40]	50.0%	90.0%
Africa[41]	18.5%	34.2%
Syria[42]	61.0%	90.1%

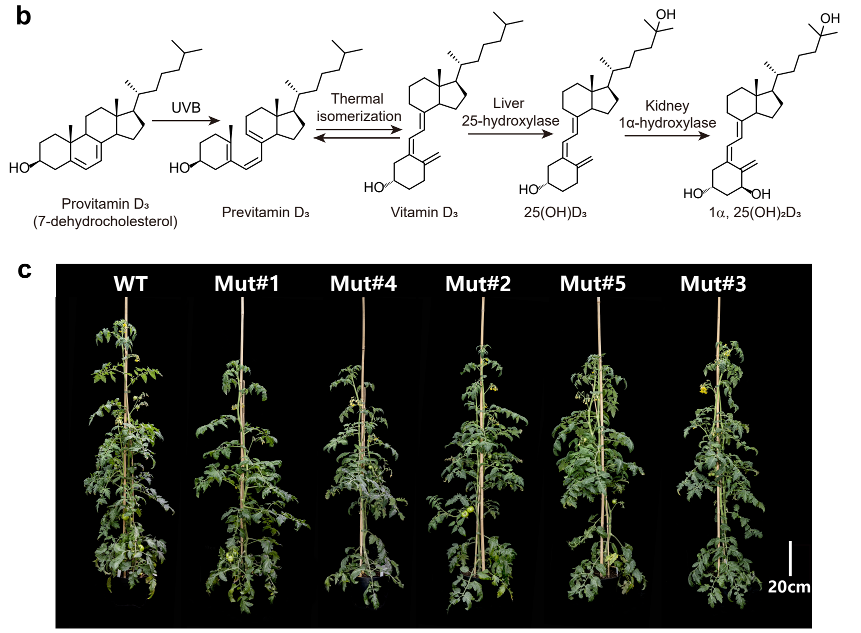

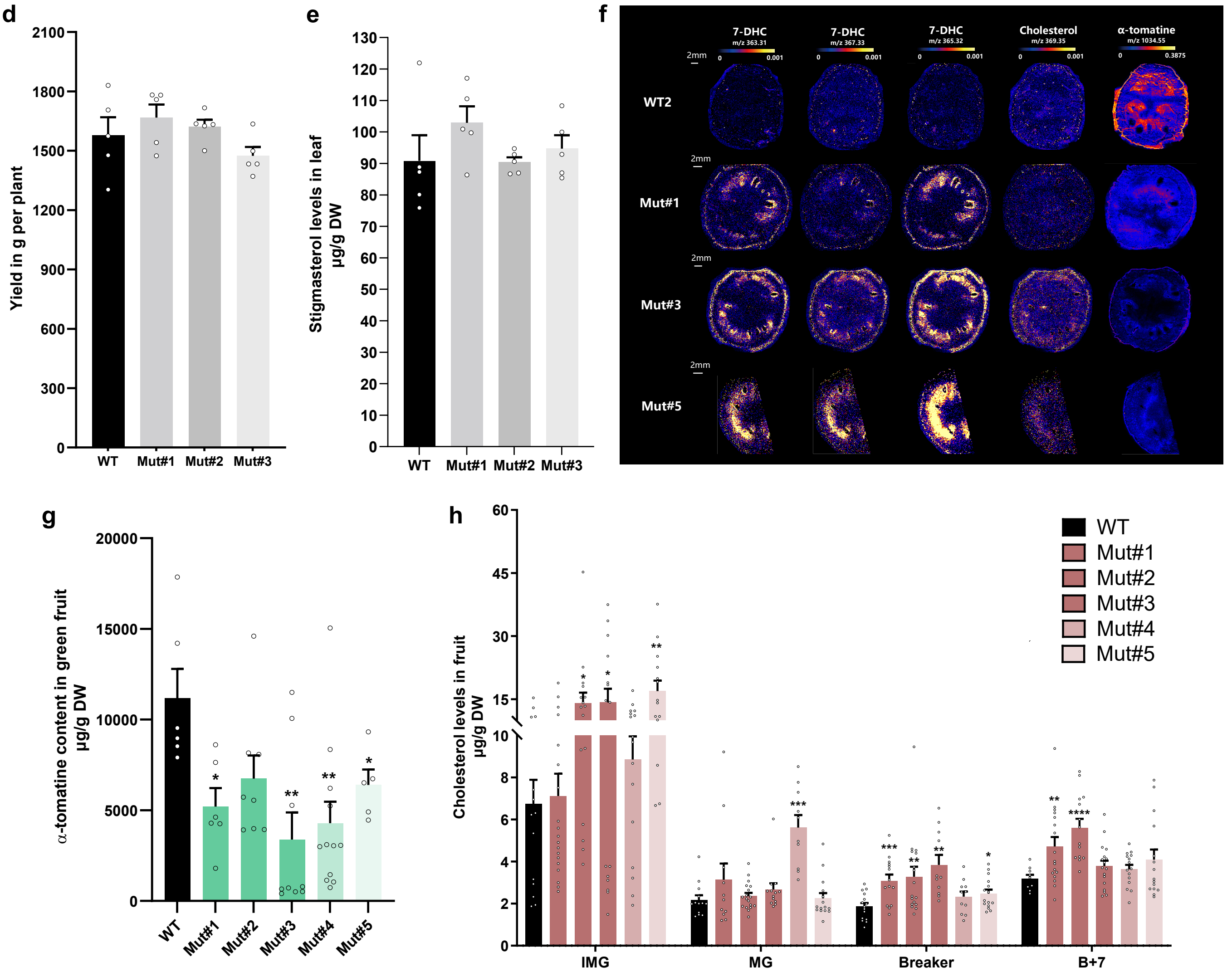

Supplementary Figure 1 **a.** Vitamin D deficiency by geographical area. Cutoffs refer to serum concentrations of 25(OH)D used to define vitamin D deficiency. 25(OH)D=25-hydroxyvitamin D. Serum concentrations of 25(OH)D lower than 25 (or 30) nmol/L and 50 nmol/L are usually regarded as vitamin D deficiency and insufficiency, respectively. * Percentage for women of reproductive age; NA, data not available. **b.** Synthesis and activation of vitamin D_3 in human. Figure adapted from Jäpelt *et al.* (ref[10]). **c.** Adult plants of wild type and *Sl7-DR2* mutants. Scale bar, 20 cm. **d.** Yield of WT and *Sl7-DR2* mutant plants in g per plant (mean ± s.e.m, n=5 plants per line). Statistical significance between WT and mutants was assessed using two-tailed t-tests. No significant differences were detected. **e.**Stigmasterol content of leaves from wild-type and *Sl7-DR2* knock-out lines (mean ± s.e.m, n=5 biologically independent leaf samples per line). Statistical significance between WT and mutants was assessed using two-tailed t-tests. No significant differences were detected. **f.** MALDI images of 7-DHC (*m/z* 367.33) and its laser-induced derivative ions (*m/z* 365.32, *m/z* 363.31), cholesterol (*m/z* 369.35) and α-tomatine (*m/z* 1034.55). Scale bar, 2 mm. The HotMetal2 colour scale indicates the range of total ion current (TIC) -normalised intensity. The same metabolite is shown with identical scale intensity for wild type and mutant samples. More details can be found in the online methods. **g.** α-tomatine content of immature green fruit of wild-type and *Sl7-DR2* knock-out lines (mean ± s.e.m, n=6, 6, 8, 9, 12 and 5 biologically independent fruit samples). Statistical significance between WT and mutants was assessed using two-tailed t-tests (*$P \leq 0.05$, **$P \leq 0.01$). **h.** Cholesterol content of wild-type (WT) and *Sl7-DR2* knock-out tomato fruit during fruit ripening (IMG, immature green; MG, mature green; Breaker: fruit turning ripe; B+7, seven days after breaker: 'ripe fruit') (mean ± s.e.m). From left to right, n = 14, 19, 16, 15, 16, 14, 13, 11, 18, 13, 10, 15, 15, 14, 17, 11, 11, 15, 9, 17, 14, 17, 15, and 15 biologically independent fruit samples. Statistical significance between WT and mutants at each stage was assessed using two-tailed *t*-tests (*$P \leq 0.05$; **$P \leq 0.01$; ***$P \leq 0.001$; ****$P \leq 0.0001$). See source data for *P* values where relevant.

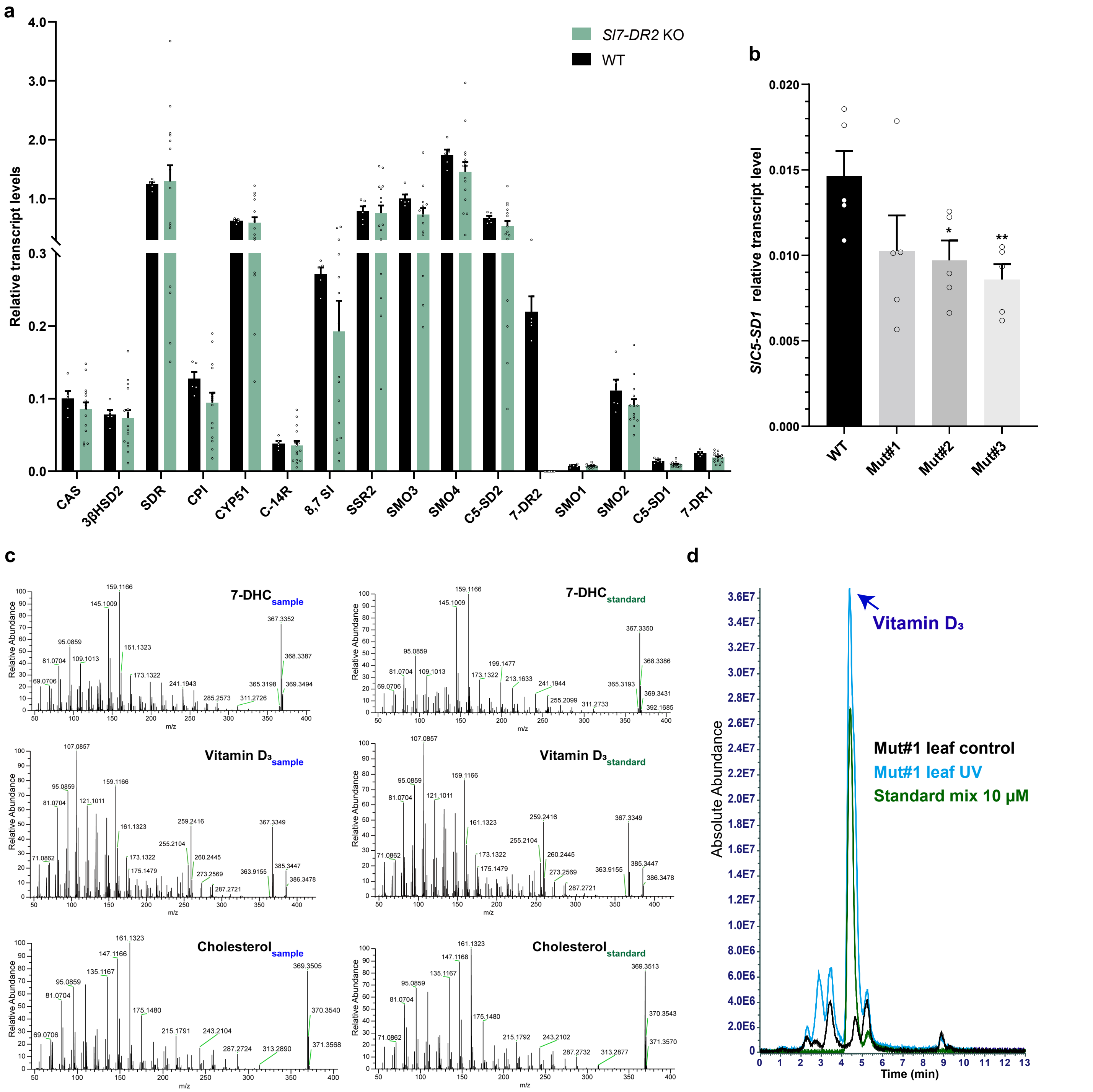

Supplementary Figure 2 **a.** Relative expression levels of genes in the cholesterol and phytosterol biosynthetic pathways in leaves of wild type and *Sl7-DR2* mutants (mean ± s.e.m). *SlActin* was used as an internal standard. WT, n=5; *Sl7-DR2* KO, n=15 (a combination of 5 samples each from Mut#1, Mut#2 and Mut#3, which carry the same mutation in *Sl7-DR2*). **b.** Relative expression levels of *SlC5-SD1* in leaves of wild type and *Sl7-DR2* mutants. *SlActin* was used as an internal standard (mean ± s.e.m, n=5 biologically independent leaf samples for each line). Statistical significance between WT and mutants was assessed using two-tailed t-tests (**P* ≤ 0.05, ***P* ≤ 0.01). See source data for *P* values where relevant. **c.** Representative LC-MS spectra of 7-DHC, vitamin D_3 and cholesterol from analysed samples and corresponding authentic standards. **d.** Overlayed chromatograms of extracts from Mut#1 leaf tissues with and without 1h UV irradiation (shown in light blue and black, respectively), as well as standard mix (10 µM of 7-DHC, vitamin D_3 and cholesterol), *m/z*= 385.3442-385.3480. Vitamin D_3 was present primarily in the UV irradiated sample.

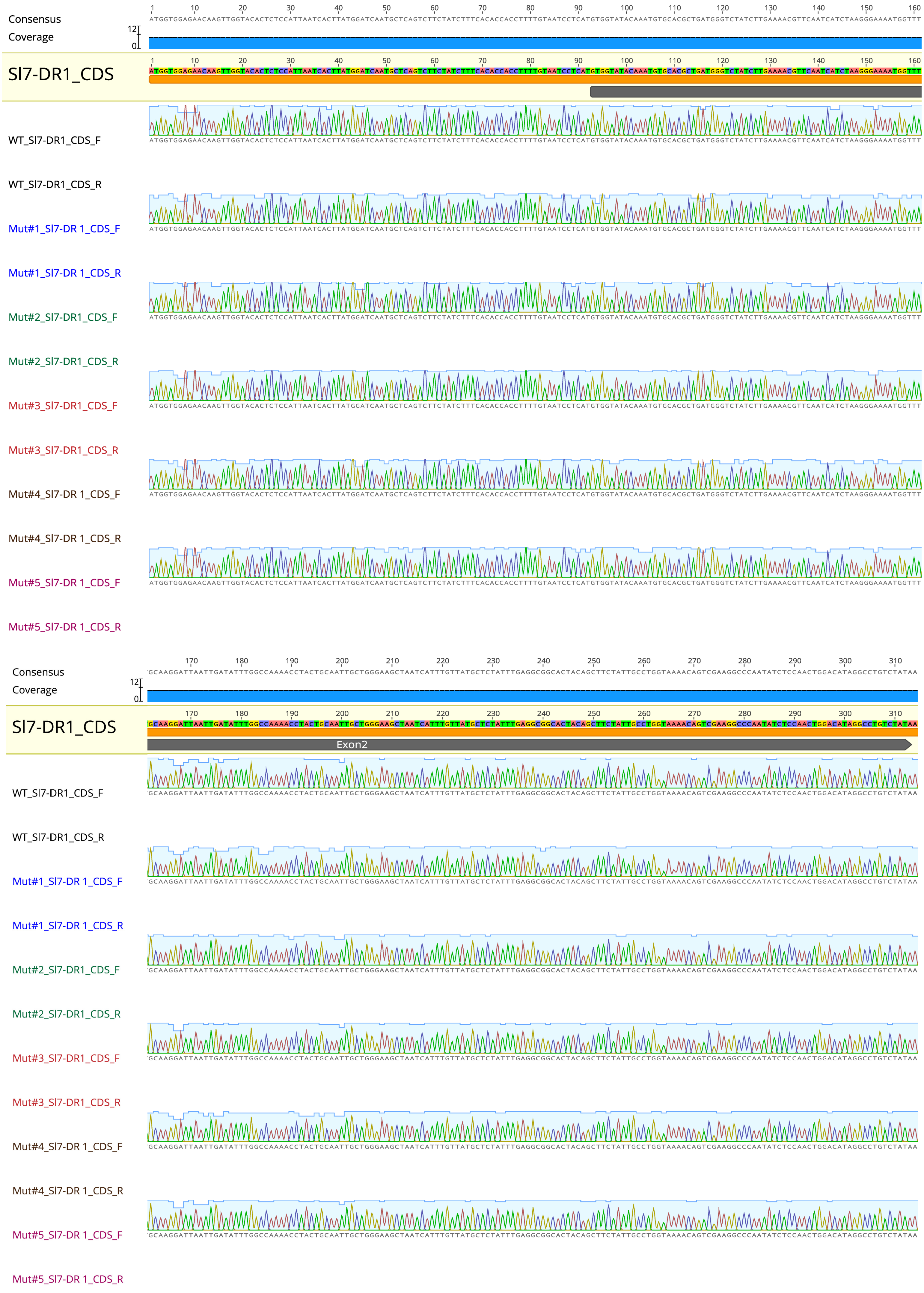
Consensus
Coverage
Sl7-DR1_CDS
WT_Sl7-DR1_CDS_F
WT_Sl7-DR1_CDS_R
Mut#1_Sl7-DR 1_CDS_F
Mut#1_Sl7-DR 1_CDS_R
Mut#2_Sl7-DR1_CDS_F
Mut#2_Sl7-DR1_CDS_R
Mut#3_Sl7-DR1_CDS_F
Mut#3_Sl7-DR1_CDS_R
Mut#4_Sl7-DR 1_CDS_F
Mut#4_Sl7-DR 1_CDS_R
Mut#5_Sl7-DR 1_CDS_F
Mut#5_Sl7-DR 1_CDS_R
Consensus
Coverage
Sl7-DR1_CDS
Exon2
WT_Sl7-DR1_CDS_F
WT_Sl7-DR1_CDS_R
Mut#1_Sl7-DR 1_CDS_F
Mut#1_Sl7-DR 1_CDS_R
Mut#2_Sl7-DR1_CDS_F
Mut#2_Sl7-DR1_CDS_R
Mut#3_Sl7-DR1_CDS_F
Mut#3_Sl7-DR1_CDS_R
Mut#4_Sl7-DR 1_CDS_F
Mut#4_Sl7-DR 1_CDS_R
Mut#5_Sl7-DR 1_CDS_F
Mut#5_Sl7-DR 1_CDS_R

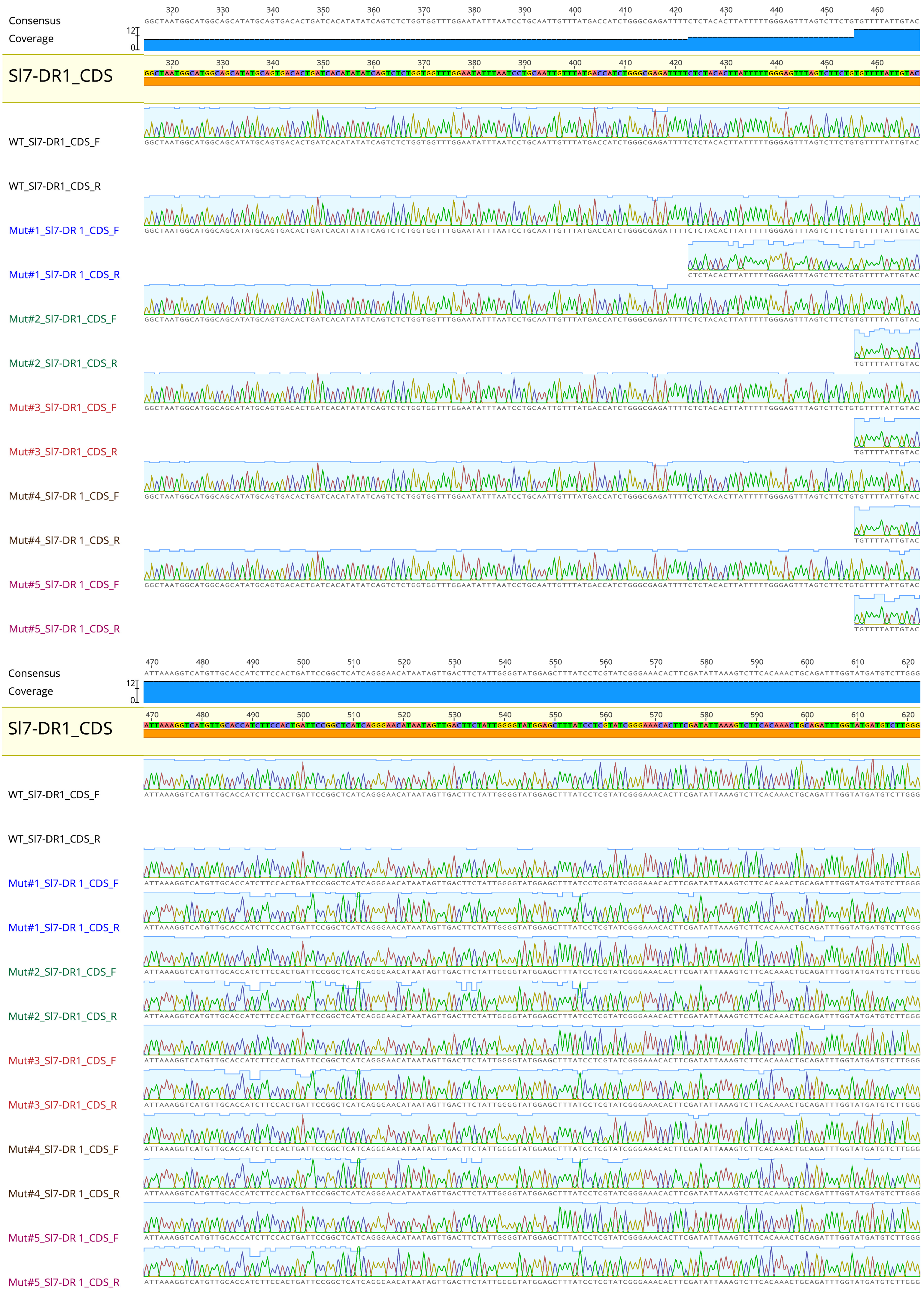
Consensus
Coverage
Sl7-DR1_CDS
WT_Sl7-DR1_CDS_F
WT_Sl7-DR1_CDS_R
Mut#1_Sl7-DR 1_CDS_F
Mut#1_Sl7-DR 1_CDS_R
Mut#2_Sl7-DR1_CDS_F
Mut#2_Sl7-DR1_CDS_R
Mut#3_Sl7-DR1_CDS_F
Mut#3_Sl7-DR1_CDS_R
Mut#4_Sl7-DR 1_CDS_F
Mut#4_Sl7-DR 1_CDS_R
Mut#5_Sl7-DR 1_CDS_F
Mut#5_Sl7-DR 1_CDS_R

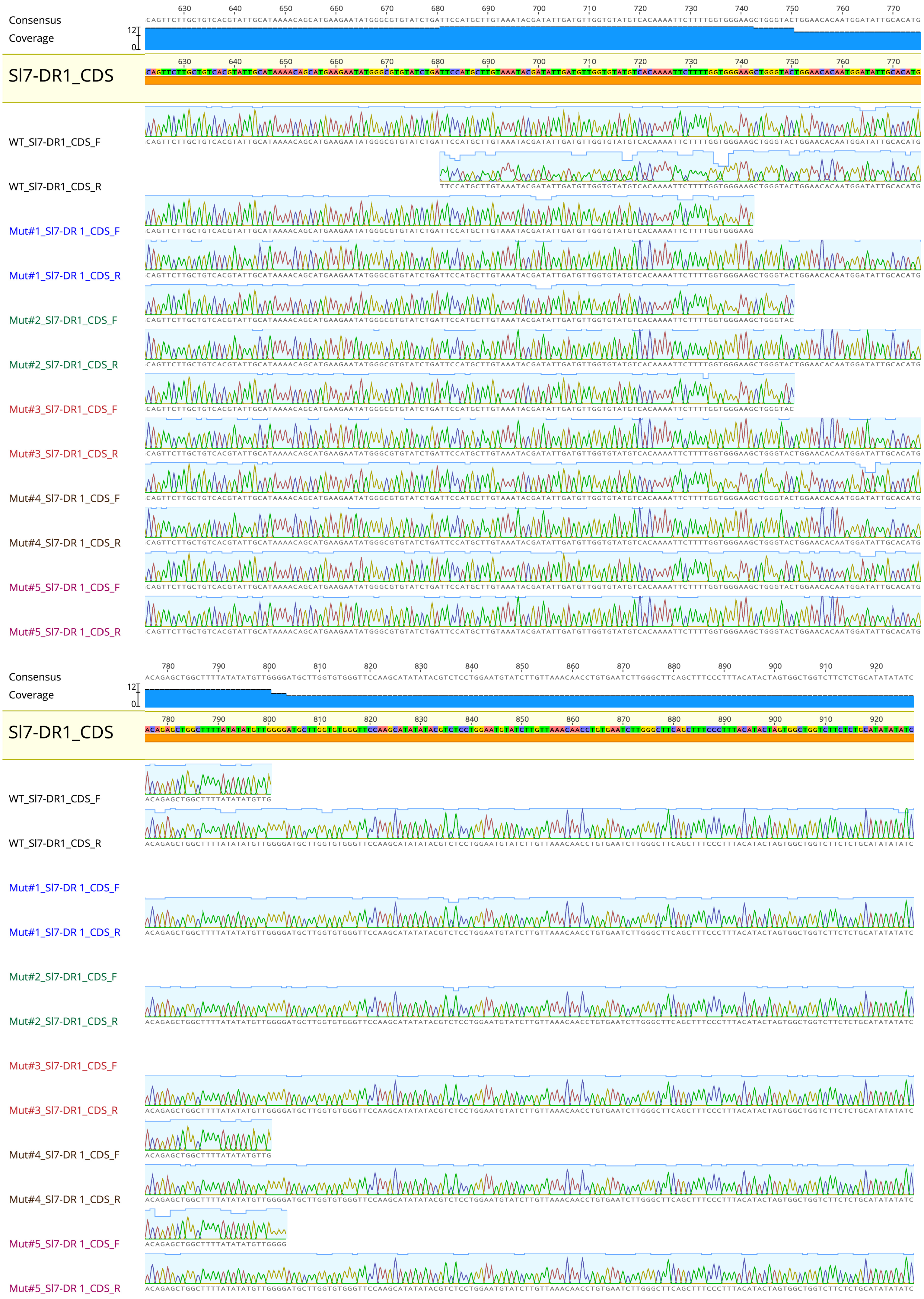

Consensus
Coverage
Sl7-DR1_CDS
WT_Sl7-DR1_CDS_F
WT_Sl7-DR1_CDS_R
Mut#1_Sl7-DR 1_CDS_F
Mut#1_Sl7-DR 1_CDS_R
Mut#2_Sl7-DR1_CDS_F
Mut#2_Sl7-DR1_CDS_R
Mut#3_Sl7-DR1_CDS_F
Mut#3_Sl7-DR1_CDS_R
Mut#4_Sl7-DR 1_CDS_F
Mut#4_Sl7-DR 1_CDS_R
Mut#5_Sl7-DR 1_CDS_F
Mut#5_Sl7-DR 1_CDS_R

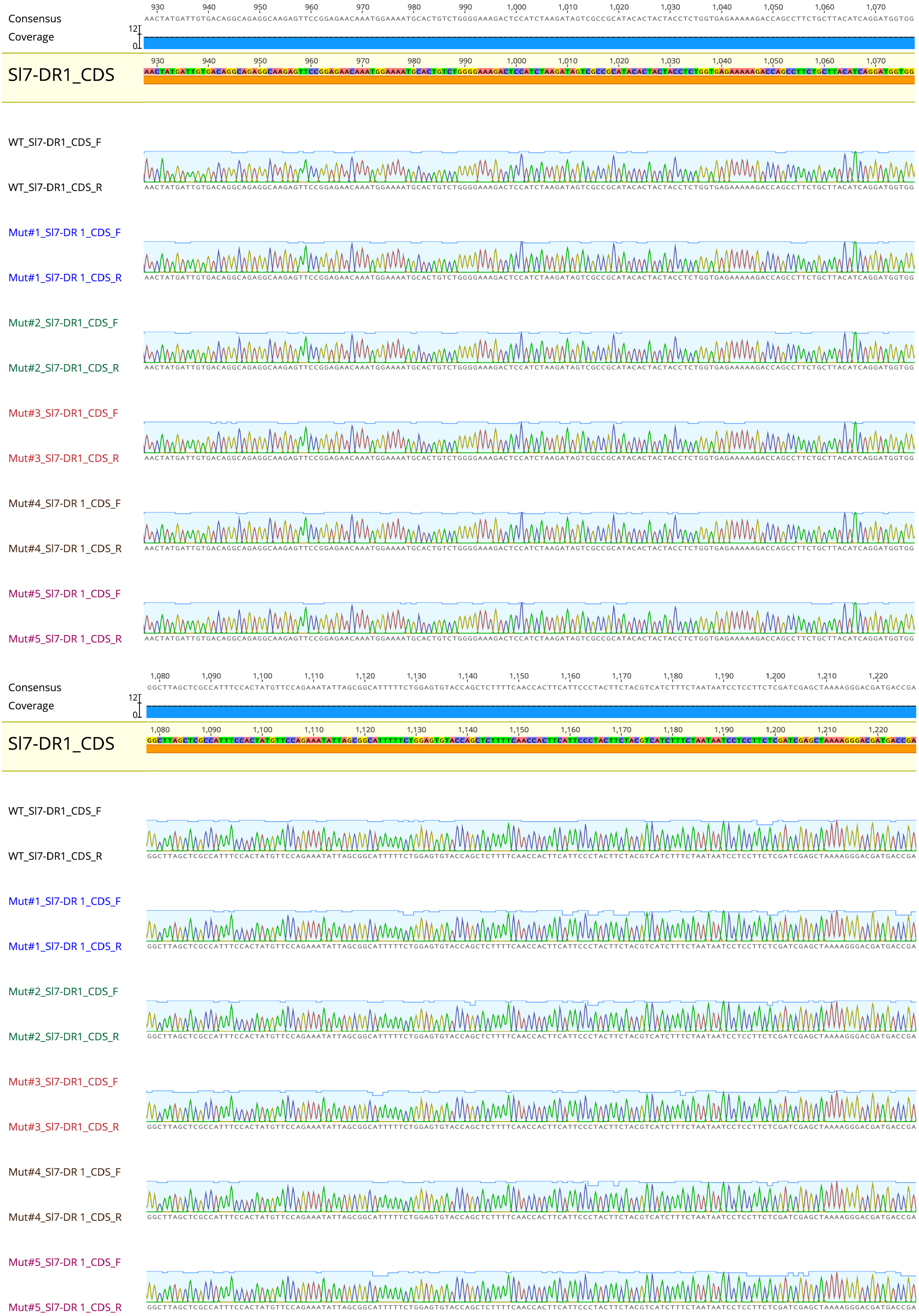
Consensus
Coverage
Sl7-DR1_CDS
WT_Sl7-DR1_CDS_F
WT_Sl7-DR1_CDS_R
Mut#1_Sl7-DR 1_CDS_F
Mut#1_Sl7-DR 1_CDS_R
Mut#2_Sl7-DR1_CDS_F
Mut#2_Sl7-DR1_CDS_R
Mut#3_Sl7-DR1_CDS_F
Mut#3_Sl7-DR1_CDS_R
Mut#4_Sl7-DR 1_CDS_F
Mut#4_Sl7-DR 1_CDS_R
Mut#5_Sl7-DR 1_CDS_F
Mut#5_Sl7-DR 1_CDS_R
Consensus
Coverage
Sl7-DR1_CDS
WT_Sl7-DR1_CDS_F
WT_Sl7-DR1_CDS_R
Mut#1_Sl7-DR 1_CDS_F
Mut#1_Sl7-DR 1_CDS_R
Mut#2_Sl7-DR1_CDS_F
Mut#2_Sl7-DR1_CDS_R
Mut#3_Sl7-DR1_CDS_F
Mut#3_Sl7-DR1_CDS_R
Mut#4_Sl7-DR 1_CDS_F
Mut#4_Sl7-DR 1_CDS_R
Mut#5_Sl7-DR 1_CDS_F
Mut#5_Sl7-DR 1_CDS_R

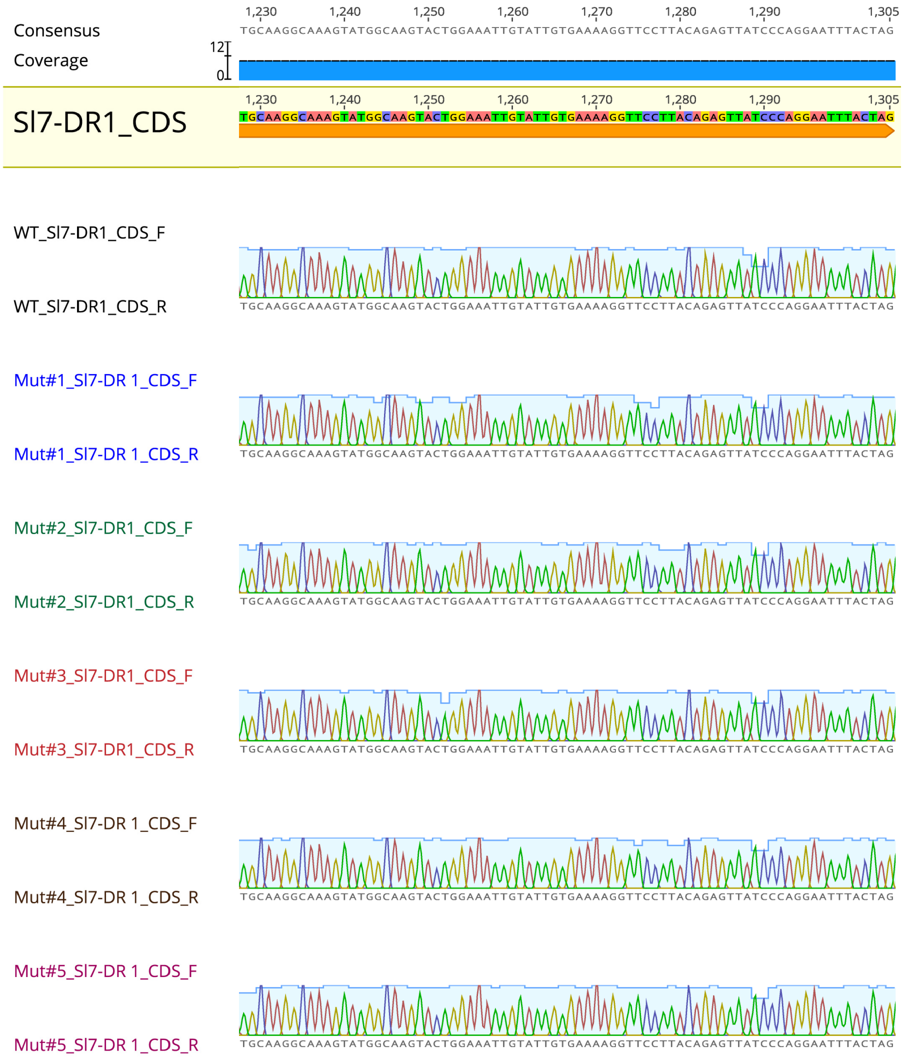

Supplementary Figure 3 Sequencing results of coding sequences (CDS) of *Sl7-DR1* amplified from cDNA of wildtype and five independent Sl7-DR2 knock-out lines. No mutations of *Sl7-DR1* were detected in any of the mutants.

Supplementary Table 1. Primers used in RT-qPCR.

Gene	Oligo	Sequence	Product Size
SlActin (*Solyc03g078400*)	ACT-q_F	GGGGGCTATGAATGCACGGT	
	ACT-q_F	GGCAATGCATCAGGCACCTC	110bp
SlSSR2 (*Solyc02g069490*)	SSR2_q_F1	TGTCATTCAAGATCTCCTTGTTCCT	
	SSR2_q_R1	CAAATTGGATATACCTCCATCTCGC	87bp
Sl7-DR1 (*Solyc01g009310*)	7-DR1_q_F1	TTGGGCTTCAGCTTTCCCTT	
	7-DR1_q_R1	AACTCTTGCCTCTGCCTGTC	88bp
SlSMO4 (*Solyc06g005750*)	SMO4_q_F1	GTCCATCATGAGTACGCGACA	
	SMO4_q_R1	ATTGCAGGACCAATGACCGT	101bp
SlSMO3 (*Solyc01g091320*)	SMO3_q_F1	TCACAGCGGGTATGAATTTCCA	
	SMO3_q_R	ACTGTGACAACTTTCCCCAACA	106bp
Sl3βHSD2 (*Solyc02g081730*)	3βHSD2_q_F	TAAAGGCCTGCTGACATGCT	
	3βHSD2_q_R	GATTTTCCAGCCCTTGCAGC	102bp
SlSDR (*Solyc01g073640*)	SDR_q_F	GCACGCGGCTAAAGTTATGG	
	SDR_q_R	GGCCCCAGCTAAAATCCCTT	85bp
SlC5-SD2 (*Solyc02g086180*)	C5-SD2_q_F	GGAAGCCTTATGGACGGCTA	
	C5-SD2_q_R	TGGTATGATAACCGGCACCC	80bp
SlCPI (*Solyc12g098640*)	CPI_q_F	CCCTAGCAAGAGATGGGGTG	
	CP1_q_R	TGGGACCACAATACCAAGGC	82bp
SlCYP51 (*Solyc01g008110*)	CYP51_q_F	CGTACAGGCAAGGCAGAGAA	
	CYP51_q_R	TCTCACCCTCTGTTGTTGGC	85bp
SlSMO1 (*Solyc08g079570*)	SMO1_q_F	GGCCAAAGCCAGAGCAATTT	
	SMO1_q_R	AGCACCCTTTAACTGCTGGA	111bp
SlSMO2 (*Solyc06g076410*)	SMO2_q_F	AGAAAGGGCAGGATGGTTGAG	
	SMO2_q_R	ATACAGCAACAGGCGAGTGA	94bp
Sl8,7 SI (Solyc06g082980)	8,7 SI_q_F	TTGCCTGGTTTTGTTCCCCT	
	8,7 SI_q_R	AACACCACAACCAGTACGGA	83bp
Sl7-DR2 (*Solyc06g074090*)	7-DR2_q_F	GAGAATGGCCTGCAAGGACTA	
	7-DR2_q_R	AGGCAACAAAAGCTGAAGTGT	111bp
SlC14-R (*Solyc09g009040*)	C-14R_q_F	CGGTGTTCAGAGGAGCCAAT	
	C-14R_q_R	GAAGCAAGCAACTTTCCCCC	106bp
SlCAS (*Solyc04g070980*)	CAS_q_F	GTATTGCTAAAGCTGCCGCC	
	CAS_q_R	GGTGAAGCAAACTGCCCAAG	86bp
SlC5-SD1 (*Solyc02g063240*)	C5-SD1_q_F	ACTCTCCCCATTTGCTGGTT	
	C5-SD1_q_R	GTGTGTGGTGAAATGCACAGG	106bp

Supplementary Table 2. Detailed information for the identification of cholecalciferol (vitamin D_3), 7-DHC and cholesterol by MALDI.

Standards spotted side by side on WT tissue section				Standards spotted on glass slides separately									m/z Observed in mutants with same typical localisation pattern			
m/z	normalised intensity	drift time (dt) (bins)	colocalised with	cholcal			7dh			chol			Mut#1		Mut#5	
				m/z	intensity	dt bins	m/z	intensity	dt bins	m/z	intensity	dt bins	m/z	dt bins	m/z	dt bins
360.3638	0.06069	12.30	7dh and chol	360.3530	1708	12.27				360.3619	607	12.26				
361.3667	0.01652	12.28					361.2889	2285	10.88							
*363.3063	0.06303	11.00	7dh	*363.305	682	10.97	*363.3047	17699	10.92				*363.3043	10.93	*363.3058	11.03
							364.3088	6183	10.95							
*365.321	0.0324	10.99	7dh	*365.3204	4144	11.08	*365.3192	15508	10.92				*365.319	10.93	*365.3182	11.01
366.3024	0.0083	11.25	7dh	366.3253	1884	11.24	366.3237	6149	11.01							
				*367.3357	11782	11.23	*367.3301	7997	11.14	*367.3384	1366	11.22	*367.3336	11.27	*367.3242	11.43
				368.3391	3171	11.24	368.3340	2587	11.18	368.3438	1037	11.39				
#369.3471	0.2623	11.74	chol	#369.3192	1713	11.39	#369.3162	4587	11.04	#369.3546	14248	11.54	#369.3381	11.78	#369.3350	11.82
370.2974	0.01066	11.27	7dh and chol							370.3576	3924	11.55				
							373.3100	1862	10.91							
^376.3227	0.02078	11.56	cholcal													
^377.3254	0.01356	11.55	cholcal													
							379.2997	6491	11.20							
380.3334	0.25962	10.42					380.3202	5738	11.33							
*381.3328	0.08153	10.68		*381.3138	1098	11.48	*381.3159	13342	11.20				*381.3266	10.79	*381.3273	11.00
382.3397	0.0398	10.80		382.3255	906	11.69	382.3328	7426	11.07				382.3340	10.77	382.3319	11.21
				383.3272	3248	11.71	383.3342	4302	11.20				383.3567	11.62	383.3497	11.78
				384.3326	1615	11.86	384.3433	3929	11.47							
				385.3326	2458	11.69										
388.3957	0.04975	13.18	7dh and chol	388.3934	931	13.26				388.3950	1023	13.19				
389.3983	0.03727	13.17	7dh and chol													

*cholcal: Cholecalciferol, vitamin D_3; 7dh: 7-dehydrocholesterol (7-DHC); chol: Cholesterol. Representative mass for 7-DHC, cholesterol and vitamin D_3 are highlighted and marked as *, # and ^, respectively.

References

31. Hilger, J. et al. A systematic review of vitamin D status in populations worldwide. *Br. J. Nutr.* **111**, 23–45 (2014).

32. Schleicher, R. L. et al. National estimates of serum total 25-hydroxyvitamin D and metabolite concentrations measured by liquid chromatography–tandem mass spectrometry in the US population during 2007–2010. *J. Nutr.* **146**, 1051–1061 (2016).

33. Sarafin, K. et al. Standardizing 25-hydroxyvitamin D values from the Canadian Health Measures Survey. *Am. J. Clin. Nutr.* **102**, 1044 –1050 (2015).

34. Cashman, K. D. et al. Vitamin D deficiency in Europe: pandemic? *Am. J. Clin. Nutr.* **103**, 1033–1044 (2016).

35. Ministry of Public Health, UNICEF, Aga Khan University. *National Nutrition Survey, Afghanistan* (2013).

36. Liu, W. et al. Vitamin D status in mainland of China: a systematic review and meta-analysis. *EClinicalMedicine* **38**, 101017 (2021).

37. Nakamura, K. et al. Impact of demographic, environmental, and lifestyle factors on vitamin D sufficiency in 9084 Japanese adults. *Bone* **74**, 10–17 (2015).

38. Cashman, K. D., Sheehy, T. & O'Neill, C. M. Is vitamin D deficiency a public health concern for low middle income countries? A systematic literature review. Eur. J. Nutr. 58, 433–453 (2019).

39. Siddiqee, M. H., Bhattacharjee, B., Siddiqi, U. R. & MeshbahurRahman, M. High prevalence of vitamin D deficiency among the South Asian adults: a systematic review and meta-analysis. *BMC Public Health* **21**, 1823 (2021).

40. Arabi, A., El Rassi, R. & El-Hajj Fuleihan, G. Hypovitaminosis D in developing countries—prevalence, risk factors and outcomes. *Nat. Rev. Endocrinol.* **6**, 550–561 (2010).

41. Mogire, R. M. et al. Prevalence of vitamin D deficiency in Africa: a systematic review and meta-analysis. *Lancet Glob. Health* **8**, e134–e142 (2020).

42. Sayed-Hassan, R., Abazid, N. & Alourfi, Z. Relationship between 25-hydroxyvitamin D concentrations, serum calcium, and parathyroid hormone in apparently healthy Syrian people. *Arch. Osteoporos.* **9**, 176 (2014).
