# Supplementary material for: Biofortified tomatoes provide a new route to vitamin D sufficiency
Source: Nat Plants. 2022 May 23;8(6):611–6. doi: 10.1038/s41477-022-01154-6 (PMC9213236; doi:10.1038/s41477-022-01154-6)
Supplement: Supplementary file 2 — Reporting Summary [file 41477_2022_1154_MOESM2_ESM.pdf]

nature portfolio

Corresponding author(s): Cathie Martin

Last updated by author(s): Apr 4, 2022

Reporting Summary

Nature Portfolio wishes to improve the reproducibility of the work that we publish. This form provides structure for consistency and transparency in reporting. For further information on Nature Portfolio policies, see our Editorial Policies and the Editorial Policy Checklist.

Statistics

For all statistical analyses, confirm that the following items are present in the figure legend, table legend, main text, or Methods section.

n/a	Confirmed	
☐	☒	The exact sample size (n) for each experimental group/condition, given as a discrete number and unit of measurement
☐	☒	A statement on whether measurements were taken from distinct samples or whether the same sample was measured repeatedly
☐	☒	The statistical test(s) used AND whether they are one- or two-sided<br>*Only common tests should be described solely by name; describe more complex techniques in the Methods section.*
☒	☐	A description of all covariates tested
☐	☒	A description of any assumptions or corrections, such as tests of normality and adjustment for multiple comparisons
☐	☒	A full description of the statistical parameters including central tendency (e.g. means) or other basic estimates (e.g. regression coefficient) AND variation (e.g. standard deviation) or associated estimates of uncertainty (e.g. confidence intervals)
☐	☒	For null hypothesis testing, the test statistic (e.g. F, t, r) with confidence intervals, effect sizes, degrees of freedom and P value noted<br>*Give P values as exact values whenever suitable.*
☒	☐	For Bayesian analysis, information on the choice of priors and Markov chain Monte Carlo settings
☒	☐	For hierarchical and complex designs, identification of the appropriate level for tests and full reporting of outcomes
☒	☐	Estimates of effect sizes (e.g. Cohen's d, Pearson's r), indicating how they were calculated

Our web collection on statistics for biologists contains articles on many of the points above.

Software and code

Policy information about availability of computer code

Data collection	All presented data have been acquired using existing and routinely used software. Primers were designed with NCBI primer BLAST tool. Quantitative real-time PCR data were collected by X96 Touch™ Real-Time PCR Detection System (Biorad). Liquid chromatographic and mass spectrometry data were collected by the Dionex UltimMate (Thermo Fisher Scientific) and Q Exactive Orbitrap Mass Spectrometer (Thermo Scientific). Optical images were taken using a Canon 5D Mark IV camera with a Canon MP-E 65mm f/2.8 1-5x Macro Photo lens (Canon Inc, Ōta, Tokyo, Japan). MALDI imaging was performed with a Synapt G2-Si mass spectrometer with a MALDI source (Waters, Wilmslow, UK) equipped with a 2.5 kHz Nd:YAG laser operated at 355 nm.
Data analysis	Bio-Rad CFX Maestro Software. Capture One photo editing software (Capture One, Frederiksberg, Denmark). High Definition Imaging (HDI)1.4. Xcalibur software (version 4.3, Thermo Scientific). GraphPad (version 9.2.0). Geneious (version 10.2.6).

For manuscripts utilizing custom algorithms or software that are central to the research but not yet described in published literature, software must be made available to editors and reviewers. We strongly encourage code deposition in a community repository (e.g. GitHub). See the Nature Portfolio guidelines for submitting code & software for further information.

Data

Policy information about availability of data

All manuscripts must include a data availability statement. This statement should provide the following information, where applicable:

- Accession codes, unique identifiers, or web links for publicly available datasets
- A description of any restrictions on data availability
- For clinical datasets or third party data, please ensure that the statement adheres to our policy

Statistical Source Data with exact P values underlying Figures 1c-d, Figures 2b-e, Supplementary Figures 1d-e, 1g-h, Supplementary Figures 2a-b and Extended Data Figure 2 are provided as Source Data. Materials generated in this study are available from the corresponding author upon request.

Field-specific reporting

Please select the one below that is the best fit for your research. If you are not sure, read the appropriate sections before making your selection.

☒ Life sciences ☐ Behavioural & social sciences ☐ Ecological, evolutionary & environmental sciences

For a reference copy of the document with all sections, see nature.com/documents/nr-reporting-summary-flat.pdf

Life sciences study design

All studies must disclose on these points even when the disclosure is negative.

Sample size	Sample sizes were chosen based on published studies, previous experience on the similar experiments in our lab and the availability of samples. At least three independent biological replicates were applied in each experiment. Sample sizes were described in figure legends where relevant. No statistical methods were used to predetermine sample size.
Data exclusions	No data were excluded from the analysis.
Replication	All the replication were successfully performed independently. Five independent transgenic lines were generated. At least three independent CRISPR-Cas9 knock-out lines were included in each analysis.
Randomization	Plants were grown in a randomized design. Sample analysis order has been further independently randomized for metabolic profiling.
Blinding	For leaf and fruit sample harvesting, corresponding metabolic profiling and MALDI imaging, samples were labeled with IDs denoting their randomised run order for both wildtype and mutant lines. Blinding was not used in the remaining experiments in this study because these kinds of experiments generally did not require blinding.

Reporting for specific materials, systems and methods

We require information from authors about some types of materials, experimental systems and methods used in many studies. Here, indicate whether each material, system or method listed is relevant to your study. If you are not sure if a list item applies to your research, read the appropriate section before selecting a response.

Materials & experimental systems

n/a	Involved in the study	
☒	☐	Antibodies
☒	☐	Eukaryotic cell lines
☒	☐	Palaeontology and archaeology
☒	☐	Animals and other organisms
☒	☐	Human research participants
☒	☐	Clinical data
☒	☐	Dual use research of concern

Methods

n/a	Involved in the study	
☒	☐	ChIP-seq
☒	☐	Flow cytometry
☒	☐	MRI-based neuroimaging

March 2021
